# The Role of Continuous Glucose Monitoring in Detecting Early Dysglycemia and Clinical Outcomes in Patients with Cystic Fibrosis

**DOI:** 10.3390/medicina60030477

**Published:** 2024-03-14

**Authors:** Lora Stanka Kirigin Biloš, Velimir Altabas, Andrea Vukić Dugac, Maja Baretić

**Affiliations:** 1 Department of Endocrinology, Diabetes and Metabolic Diseases “Mladen Sekso”, University Hospital Centre “Sestre Milosrdnice”, 10000 Zagreb, Croatia; 2 School of Medicine, University of Zagreb, 10000 Zagreb, Croatia; andrea.vukic.dugac@kbc-zagreb.hr; 3 University Hospital Centre Zagreb, Cystic Fibrosis Centre for Pediatrics and Adults, 10000 Zagreb, Croatia; maja.baretic@kbc-zagreb.hr; 4 Clinic for respiratory diseases “Jordanovac”, University Hospital Centre Zagreb, 10000 Zagreb, Croatia; 5 Internal clinic, Department of Endocrinology and Diabetology, University Hospital Centre Zagreb, 10000 Zagreb, Croatia

**Keywords:** cystic fibrosis-related diabetes, continuous glucose monitoring, screening, clinical outcomes

## Abstract

Cystic fibrosis-related diabetes (CFRD) is the most common comorbidity in patients with cystic fibrosis (CF). CFRD has been correlated with important clinical outcomes, including poor nutrition, reduced pulmonary function, and earlier mortality. However, clinical decline due to abnormalities of blood glucose (dysglycemia) begins early in CF, before the diagnosis of CFRD by the gold-standard oral glucose tolerance test (OGTT). Continuous glucose monitoring (CGM) has been validated in patients with CF and has been recognized as a valuable tool in detecting early glucose abnormalities in patients with CF. Several CGM parameters have been used to predict CFRD in some but not all studies, and there is no consensus regarding CGM use for diagnostic purposes. Thus, it remains a complementary test to OGTT in CFRD diagnosis. The aim of this review is to provide an update on the pathophysiological mechanisms of CFRD, recent advances in the use of CGM for CFRD screening, and the association between CGM measures and CF-related clinical outcomes.

## 1. Introduction

Cystic fibrosis is the most prevalent autosomal recessive genetic condition caused by mutations in the cystic fibrosis transmembrane conductance regulator (CFTR) protein, which regulates chloride and bicarbonate transport in epithelial cells [1]. CFTR gene mutations can result in a wide range of CFTR protein defects, affecting its synthesis, folding, function, regulation, and half-life. Over 2000 mutations have been identified so far. This large number of gene variants are classified into seven classes (including one class for the complete absence of CFTR) resulting in diverse clinical manifestations. CFTR dysfunction results in the accumulation of thick, sticky mucus in several organs, such as the lungs, pancreas, intestine, and liver [2].

Progressive respiratory impairment is the main contributor to health complications, resulting in respiratory failure, which is the main cause of premature death. The thick, viscous secretions can also block pancreatic ducts, contributing to exocrine glandular insufficiency, resulting in the malabsorption of fats and proteins and subsequent malnutrition and growth failure. Therefore, pulmonary and nutritional status are important markers of overall health in patients with CF [3].

Over the last decade, the life expectancy of patients with CF has dramatically increased, mostly due to the introduction of CFTR modulators, which partially restore CFTR function. However, this improvement has led to a growing incidence of non-pulmonary complications, including cystic fibrosis-related diabetes [4]. Cystic fibrosis-related diabetes (CFRD) occurs in up to 20% of adolescents and 30% to 50% of adults with CF [5] and adds an additional burden to an already complicated disease. The rising prevalence also requires diabetologists to acquaint themselves with the pathophysiology and management of a once-rare form of diabetes [6].

The onset of CFRD is insidious, and screening is crucial. The development of CFRD has been associated with worse clinical outcomes, including pulmonary and nutritional decline, increased pulmonary exacerbations, and increased mortality [7,8]. This could be explained by insulin deficiency, which is a catabolic state [9], and hyperglycemia itself, which is associated with an increased risk of infection. Patients with CFRD also have elevated glucose levels on their airway surfaces, which may promote bacterial overgrowth, and a less effective inflammatory response [10,11].

Insulin is currently the primary treatment modality for CFRD and possibly prediabetes; however, CFRD is a heterogeneous disease, and not all patients with CFRD need insulin therapy. Insulin has effects beyond blood glucose control, including improving respiratory function and enhancing body mass index (BMI) by repleting muscle mass and protein reserves [12,13]. Several insulin regimens are available, and the choice of regimen should be based on the patient’s blood glucose variability and disease severity (e.g., basal insulin only, multiple daily injections like basal–bolus therapy, or an insulin pump). The optimal regimen for patients with CFRD is still debated, but patients with CFRD typically require less insulin compared to those with type 1 diabetes [4,14]. Oral antidiabetic agents, as well as non-insulin injectable therapies, have been investigated in CFRD but are generally not recommended due to their lower efficacy and/or side effects. Nutritive support is of utmost importance in the treatment of CFRD. As opposed to patients with type 1 and type 2 diabetes, optimal management for usually undernourished CF patients involves a high-caloric diet, aiming for a caloric intake 20–100% higher than that of healthy peers [11,12]. Physical activity is useful in maintaining blood glucose control and reducing insulin requirements but may be accompanied by an increased risk of hypoglycemia [12,15].

Management of CFRD with insulin and tight glycemic control improves the clinical outcomes and life expectancy of patients with CF [16]. Therefore, in order to detect CFRD early, and apply therapeutic interventions, a yearly CFRD screening test is recommended. CFRD diagnosis using the oral glucose tolerance test (OGTT) has been associated with important CF outcomes, including poor nutrition, decreased BMI, reduced pulmonary function, and earlier mortality [17,18,19]. Compared to the OGTT, hemoglobin A1c (HbA1c) has poor sensitivity in diagnosing CFRD. Thus, the American Diabetes Association (ADA) and the CF Foundation recommend a yearly OGTT for all patients with CF aged ≥ 10 years, with the same OGTT thresholds for diabetes as in the general population [20]. Despite these recommendations, screening rates remain low (25–50% of patients get screened annually) [21], and many barriers to testing exist [4]. Furthermore, clinical decline due to dysglycemia begins early in CF, prior to CFRD diagnosis, and is also linked to pulmonary and nutritional decline [4,22]. Dysglycemia identified using CGM has been associated with worse lung function, even in patients with normal OGTT [23,24,25,26]. Therefore, other screening methods are being investigated that can not only detect CFRD, but also predict clinical decline, and intermittent continuous glucose monitoring (CGM) shows promise.

Continuous glucose monitoring devices sample glucose in the interstitial fluid, with readings every few minutes, and provide valuable information about glycemic variability and trends over time. Although CGM is typically used to optimize behavioral and pharmacological treatment in diabetes, its role in CFRD screening is increasingly being investigated [27]. CGM has been validated in patients with CF, and CGM-derived average glucose is strongly correlated with HbA1c in adults with CF [22,26]. Glycemic variability refers to the degree of fluctuation in an individual’s blood glucose levels over a set period, reflecting variations between hyperglycemia and hypoglycemia [28]. CGM data have shown that early CFRD is marked by short postprandial glycemic excursions that are often followed by reactive hypoglycemia. This means that any measure of average glucose, including HbA1c, is going to miss the glycemic variability seen in early CFRD [22,29]. Several studies have shown that CGM measures of hyperglycemia and glucose variability correlate with lung function, BMI, and infection rates in patients with CF [29,30].

Although early glucose abnormalities are commonly detected by CGM, the relationship between these CGM abnormalities and clinically relevant CF outcomes has not been fully characterized. Several CGM measures have been used to predict CFRD in some but not all studies, and there is no consensus regarding CGM use for diagnostic purposes. A key challenge in implementing CGM for screening is the lack of defined thresholds for diagnosing CFRD and initiating insulin [27]. This review will describe the pathophysiological mechanisms involved in CFRD and provide an update on the use of CGM for CFRD screening and the association between CGM parameters and CF-related clinical outcomes.

A search pertaining to the role of CGM in CFRD screening was conducted from 29 November 2023 to 31 January 2024. Human studies, reviews, and meta-analyses from the preceding 10 years that evaluated the role of CGM in detecting prediabetes, dysglycemia, CFRD, and clinical outcomes in patients with CF were examined. Online databases (MEDLINE and Web of Science) were searched using the following terms: “CGM and CFRD”, “CGM and dysglycemia in CF”, “CGM and prediabetes in CF”, and “CGM and CF outcomes”. Additionally, relevant ongoing studies registered on ClinicalTrials.gov were reviewed.

## 2. Pathophysiological Mechanisms of CFRD

Despite the increasing prevalence of CFRD, its etiology and risk factors are still poorly understood [3]. While CFRD shares characteristics with both type 1 and type 2 diabetes, it represents a distinct and unique form of diabetes with its own clinical and pathophysiological features [31]. It is primarily characterized by insulin deficiency due to pancreatic β-cell dysfunction or loss, but, unlike type 1 diabetes, there is no underlying autoimmune process [6]. As mentioned earlier, pancreatic duct obstruction contributes to chronic pancreatitis, loss of acinar tissue, ductal fibrosis, and eventually fatty transformation of pancreatic tissue and glandular insufficiency. In this way, the gradual pancreatic exocrine dysfunction damages the neighboring pancreatic islets, decreasing pancreatic hormone production [11]. The initial stage in the development of hyperglycemia involves a delay in first-phase insulin secretion, followed by a decrease in total insulin secretion [6].

However, animal models of CF show functional pancreatic β-cell defects early, even in the absence of structural pancreatic abnormalities [32], and autopsy studies reveal decreases in β-cell mass, irrespective of pancreatic exocrine disease [33]. Therefore, it is still unclear if CFTR mutations have direct effects on β-cell function. CFTR gene mutations make β-cells more susceptible to oxidative stress, which can result in apoptosis and worsen glucose control. Similar to type 2 diabetes, amyloid particles may accumulate in β-cells, leading to their malfunction [3].

Several risk factors have been associated with the development of CFRD including organ insufficiency (lung, liver, exocrine pancreas), growth delay, sex (female), residual CFTR function-related factors (residual function, genotype, genetic modifiers), history of solid organ transplantation, abnormal glucose tolerance (impaired fasting glucose, impaired glucose tolerance, indeterminate), and medication use (corticosteroids, calcineurin inhibitors) [12].

CFTR gene mutations may also diminish glucagon release from pancreatic α-cells, resulting in an insufficient response to hypoglycemia. Therefore, hypoglycemia in CF may be a result of delayed first-phase insulin secretion, glucagon deficiency, undernourishment, gastrointestinal disorders, liver disease, and incretin dysregulation [34,35].

Another important factor contributing to the onset of CFRD, related to β-cell dysfunction, is the incretin axis. Incretins, such as glucose-dependent insulinotropic peptide (GIP) and glucagon-like peptide 1 (GLP-1), are released from the gut in response to carbohydrates. These hormones stimulate insulin release into the circulation, inhibit pancreatic glucagon and somatostatin secretion, delay gastric emptying, and suppress appetite. There is some evidence that patients with CFRD have significantly lower levels of GIP and GLP-1 when compared to healthy controls [12,36]. Therefore, the secretion of incretins and the responsiveness to incretins is diminished in the pancreatic insufficiency that arises in CF [4].

**Figure 1:**
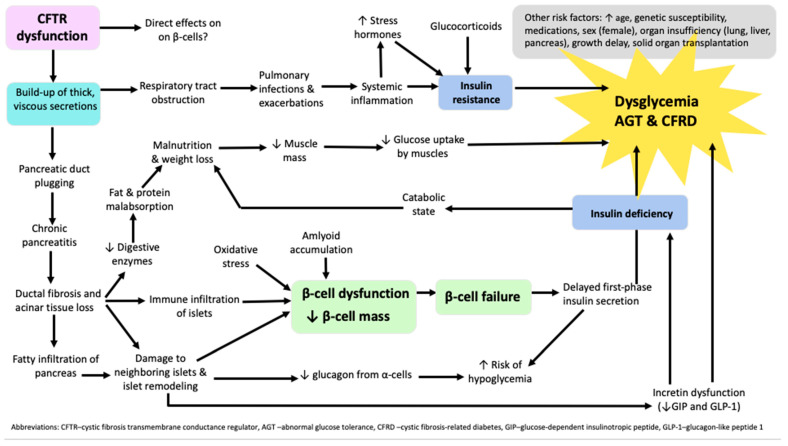
Algorithm of pathophysiological mechanisms leading to dysglycemia, abnormal glucose tolerance, and cystic fibrosis-related diabetes in patients with cystic fibrosis.

Systemic inflammation, due to respiratory infection, is another contributing factor in the development of CFRD, through its mediators like interleukin (IL)-1 beta, IL-6, CXCL10, tumor necrosis factor (TNF)-alpha, and interferon (IFN)-gamma. Inflammation leads to pancreatic damage and the immune infiltration of other islet components and also leads to insulin resistance, resulting in altered glucose tolerance and CFRD [36,37]. Hepatic insulin resistance and unsuppressed gluconeogenesis lead to increased hepatic glucose production and dysglycemia. Reduced muscle mass affects glucose uptake negatively and also contributes to hyperglycemia. Furthermore, pulmonary exacerbations, severe chronic lung disease, and glucocorticoids may aggravate insulin resistance [8]. Pathophysiological mechanisms leading to dysglycemia, abnormal glucose tolerance (AGT), and CFRD are depicted in Figure 1.

## 3. Continuous Glucose Monitoring in CFRD Screening

CFRD screening is crucial for early detection, development of personalized management strategies, and prevention of complications. Thus, determining whether CGM measures can reliably identify patients with CFRD has been a topic of great interest. While CGM is increasingly utilized for CFRD screening in many CF centers [27,38], standardized diagnostic criteria for CGM have not been established for patients with CF or any other diabetes population. The use of CGM for screening has many potential benefits and may improve screening rates in patients with CF. This screening method can be incorporated during routine clinical visits, and placing a sensor is easy and convenient, providing a more comprehensive picture of glucose abnormalities in real-life settings.

### 3.1. CGM Screening Studies

A recent prospective observational study by Scully et al. of 77 patients with CF found that CGM cut-offs of 17.5% time > 7.8 mmol/L and 3.4% time > 10.0 mmol/L had sensitivities of 87% and 90%, respectively, and specificities of 95% for identifying CFRD [22]. Patients had two blinded CGM acquisitions for up to 14 days, 3 months apart. Interestingly, both areas under the curves (AUCs) and the percentages of patients correctly classified were higher for multiple CGM measures, including average glucose (AG), standard deviation (SD), and %time metrics (%time > 7.8, >10.0, >13.9 mmol/L), than for HbA1c. However, CGM measures could not reliably distinguish patients with AGT from those with normal glucose tolerance (NGT), suggesting that CGM may not be a sensitive screening test to distinguish more milder degrees of glucose intolerance. Because the study was not originally designed as a screening study, patients with already established CFRD were included, the majority of whom were treated with insulin, which likely impacted analyses establishing cut-offs for CFRD. Therefore, in order to test these glycemic cut-offs, a validation analysis was performed on 22 patients who were not included in the original cohort. These were patients without CFRD or with early CFRD not yet on insulin. In this validation analysis, multiple CGM measures of hyperglycemia (%time > 7.8, >10.0, >13.9 mmol/L) all had sensitivities and negative predictive values exceeding 90% and specificities and positive predictive values exceeding 80% for identifying CFRD [22].

In contrast, a study by Chan et al. of 85 patients aged 6–25 years found that CGM (3–7 days) did not perform as well and could not reliably distinguish patients with and without CFRD [39]. In patients with CF, CGM measures, including mean sensor glucose, peak glucose, measures of hyperglycemia (%time > 7.8 mmol/L, %time > 10.0 mmol/L, %time > 11.1 mmol/L), and measures of glucose variability, including SD, coefficient of variation (CV), and mean amplitude of glycemic excursions (MAGE), had receiver operating characteristic (ROC) AUCs ranging from 0.43 to 0.57 for prediabetes and 0.47 to 0.6 for CFRD [39]. Therefore, although these results are promising, the exact CGM measures and thresholds required for CFRD diagnosis to guide therapeutic interventions need more study. Importantly, the studies by Chan et al. [39] and Scully et al. [22] were not designed as screening studies and instead evaluated whether CGM measures of hyperglycemia and glycemic variability could distinguish patients with and without OGTT-defined diabetes. The results of the aforementioned studies are presented in Table 1.

Despite the conflicting results between the aforementioned studies, they provide the most thorough evidence on the associations between CGM measures, OGTT, and CF outcomes [40]. Further studies are needed to test these CGM screening thresholds in larger validation samples. Validation samples should include patients with a range of racial and ethnic representation and different classes of CFTR mutations. In addition, the same type of CGM device should be used to increase the precision of CGM data.

In 2022, a systematic review by Kumar et al. assessed the relative risk of an arbitrary CGM diagnosis of diabetes compared to an OGTT diagnosis [40]. Studies reporting concurrent CGM measures and OGTT results were included. OGTT outcomes were categorized as NGT, AGT (indeterminate or impaired), or CFRD according to ADA criteria. CGM results were categorized as hyperglycemia (≥1 peak sensor glucose ≥ 11.1 mmol/L), dysglycemia (≥1 peak sensor glucose ≥ 7.8–11.0 mmol/L), or normoglycemia (<7.8 mmol/L). CGM hyperglycemia in those with NGT or AGT was considered an arbitrary CGM-CFRD diagnosis. The authors reported that the relative risk of an arbitrary CGM-CFRD diagnosis compared to OGTT-defined CFRD was 2.92, concluding that a single CGM reading >11.1 mmol/L is not a suitable cut-off for CFRD [40].

### 3.2. CGM to Predict Future CFRD

Hyperglycemia detected by CGM may also identify patients at risk of eventual progression to CFRD [29,41,42,43], although this has not been validated in larger prospective studies. The ProspeC-F multicenter observational study (ClinicalTrials.gov NCT05099939) is currently ongoing to investigate which CGM measures (using CGM three times apart over a period of 2 years) are the most strongly associated with CFRD diagnosis during 3 years of follow-up [44]. These results may clarify whether CGM measures can predict the evolution of dysglycemia and CFRD.

Patients with CF have various epidemiological, genetic, therapeutic, and clinical features that may impact glycemia and lead to bidirectional changes in glycemic status, which have been observed using both OGTT and CGM [12,35,40]. In addition, β-cell responsiveness is a dynamic process [45], which makes it difficult to define dysglycemia, irrespective of the screening test used. This may explain the conflicting results reported to date [22,39,40]. Navigating these challenges, while acknowledging the prognostic potential of CGM, is a challenge that all CF care teams face.

### 3.3. Reproducibility of CGM Measures for Screening

Before CGM can be clinically applied as a screening method, repeated CGM acquisitions must yield consistent and accurate results, as the lack of reproducibility has undermined confidence in the OGTT [46]. Therefore, when selecting CGM measures for future screening studies, it is essential to consider those that have demonstrated good stability over time.

CGM measures that have been shown to be stable over time include SD, CV, %time > 10 mmol/L, and %time > 13.9 mmol/L [22,26,47]. In contrast, %time > 7.8 mmol/L significantly varied in the previously mentioned study by Scully et al. between two study visits [22], suggesting that fluctuations of this milder measure of hyperglycemia may happen more often, making it less effective as a diagnostic threshold. A study by Kutney et al. assessed the reproducibility of in-home CGM screening in 29 adults and adolescents with CF not treated with insulin using two consecutive CGM acquisitions. Glycemic data from two 14-day periods were compared using published thresholds to define abnormality: ≥4.5%time > 7.8 mmol/L, >17.5%time > 7.8 mmol/L, and >3.4%time > 10 mmol/L. The threshold of ≥4.5%time > 7.8 mmol/L showed the greatest discrepancy between CGM1 and CGM2 (5 of 20 subjects had conflicting results). For the other two thresholds (>17.5%time > 7.8 mmol/L and >3.4%time > 10 mmol/L), only 1 of the 20 subjects displayed conflicting results, supporting the stability of these CGM measures [46]. Although several other studies have compared glycemic measures in repeated CGM, most had longer intervals between acquisitions (up to 1 year), which may reflect changes in the patient’s glycemic status rather than lack of reliability [48]. The previously mentioned study by Kutney et al. had more similar mean glucose values in CGM1 and CGM2, which is likely a result of the shorter interval between acquisitions [46,47].

### 3.4. Shortcomings of the OGTT for Screening

When examining CGM screening studies, it is important to point out several shortcomings of the current gold-standard OGTT-based CFRD definition to which CGM is being compared. Firstly, the OGTT-based CFRD criteria were adapted from populations at risk for type 2 diabetes [49] and were originally developed to evaluate the risk for microvascular complications, which are not the leading cause of morbidity in patients with CF. Thus, the current OGTT thresholds may not be sufficiently sensitive to assess hyperglycemia-associated nutritional and/or pulmonary function decline [50], and many experts have recommended lowering the current thresholds for intervention [51].

As already mentioned, glycemic categories based on OGTT results have shown great variability and lack reproducibility [47,52]. CFRD exists within a spectrum of glucose tolerance abnormalities from NGT, indeterminate (INDET), impaired fasting glucose (IFG), and impaired glucose tolerance (IGT) to CFRD [31]. Elevated mid-OGTT glucose loads (>11.1 mmol/L) at 30, 60, and 90 min are termed INDET, and emerging evidence suggests that INDET glycemia may be more predictive of clinical decline than 2 h levels, particularly pulmonary function decline. INDET glycemia has also been identified as a predictor of CFRD [53,54]. Abnormal glucose tolerance encompasses all glucose tolerance categories apart from NGT and includes INDET. However, not all studies have included INDET values, possibly underestimating glucose abnormalities in OGTT results [55].

### 3.5. Advantages and Disadvantages of CGM for Screening

The use of CGM for CFRD screening has potential advantages and limitations compared to the OGTT. One limitation is the lack of standardization in CGM acquisition and reporting. Different studies have published data using various CGM devices for different durations and in different settings [55]. CGM readings may vary depending on whether patients are blinded to CGM results or not. Non-standardized CGM acquisitions also mean that glucose data may be influenced by diet and activity. While it may be burdensome for patients to record dietary intake during CGM wear, this could help clarify if a high carbohydrate load could be contributing to dysglycemia. For these reasons, some authors have suggested capturing CGM readings under standardized conditions, such as with a standardized meal or glucose load for a set duration.

This was implemented in a recent study by Kutney et al. that examined the utility and reproducibility of an at-home mixed meal tolerance test (MMTT) to establish standardized CGM glycemic data in patients with CF not treated with insulin [46]. Participants underwent an MMTT (Boost High Protein Vanilla, Nestle) on day 5 of each 14-day CGM wear, and peak glucose and 2 h glucose thresholds were compared. Few participants had 2 h glucose > 7.8 mmol/L. Peak glucose > 7.8 mmol/L, >10.0 mmol/L, and >11.1 mmol/L were more common, with 10–37% of patients showing discordant MMTT CGM results. Although peak glucose showed variability between the two CGM sessions, patients with a history of abnormal OGTT displayed similar glycemic curves, suggesting that an at-home MMTT could be useful if clinically relevant thresholds were defined [46].

In comparison to CGM, OGTT testing and results are standardized but require fasting and the collection of multiple venous samples over two hours, necessitating trained personnel to conduct the test. Low gastric tolerability also hinders test adherence [35]. Continuous glucose monitoring does not require fasting, and once sensors are in place, patients can continue their daily activities without additional blood draws. Thus, CGM is more easily performed in daily practice in comparison to the OGTT. Given the substantial disease burden associated with CF, along with numerous competing clinical assessments, it is crucial to consider patient satisfaction and the acceptability of screening tests. At present, intermittent CGM appears to be well-tolerated and may be preferred over OGTT by patients [55]. Conducting studies to evaluate the acceptability and feasibility of various screening tests among both patients and CF teams is imperative.

Growing evidence suggests that CGM peaks are not as useful in CGM and that %time metrics are more helpful and should be reported. Using singular glucose peaks as CGM thresholds might amplify the effect of non-standardized conditions, whereas %time metrics may be less susceptible. Prolonged CGM duration or multiple CGM acquisitions could also alleviate some of the issues of non-standardization. Although most studies have used CGM during periods of stable disease, the earliest signs of β-cell dysfunction might be evident during acute illnesses, such as pulmonary exacerbations [40].

## 4. Continuous Glucose Monitoring and CF-Specific Clinical Outcomes

The OGTT is the gold-standard screening test in CF because it has been associated with important clinical outcomes [20]. While early dysglycemia is frequently identified by CGM and has shown correlations with poorer pulmonary and nutritional status, as well as an increased risk of pseudomonas colonization, these observations are based on small studies with limited follow-ups [8,25,42,43,56].

In 2010, Hameed et al. found that ≥4.5%time > 7.8 mmol/L predicted a decline in weight standard deviation with 89% sensitivity and 86% specificity [57]. Several other studies have attempted to find CGM measures predictive of BMI and pulmonary function decline in patients with CF, reporting that peak glucose or excursions above 11.1 mmol/L may predict pulmonary function decline, but exact thresholds for intervention have not been identified [39,42]. In the already mentioned study by Scully et al., CGM-derived measures of hyperglycemia and glucose variability correlated with BMI and forced expiratory volume in 1 s (FEV1) more strongly than HbA1c, with %time > 7.8 mmol/L showing the strongest association with FEV1 [22]. In a longitudinal prospective study involving 39 patients (ages 10–20 years) without CFRD at baseline with a mean follow-up of 3.1 years, Zorron et al. investigated whether CGM measures of hyper- and hypoglycemia could predict CFRD and/or clinical impairment (decline in BMI and/or FEV1). Patients had blinded CGM for up to 3 days, and the numbers of peaks (≥7.8 mmol/L and ≥11.1 mmol/L) and valleys (<3.0 mmol/L) were adjusted for CGM duration. Although CGM could detect glucose abnormalities not detected by OGTT and CGM-defined dysglycemia (peaks > 7.8 mmol/L) was able to identify early BMI decline, none of the studied measures predicted progression to CFRD or were associated with FEV1 [35]. However, this study used CGM cut-off points based on arbitrary OGTT cut-off points.

Recently, Declercq et al. examined the association between blinded CGM circadian glycemic patterns at night (7 days) and clinical outcomes in 47 patients (26 children, 21 adults) with IGT and/or HbA1c > 5.5% without prior CFRD. In this cohort, 96% of CGM data showed glucose values >7.8 mmol/L ≥ 4.5% of the time and at least one ≥11.1 mmol/L peak. Although no associations were found in the pediatric cohort, in adults, an AUC > 7.8 mmol/L and %time > 7.8 mmol/L during the night (10 p.m.–6 a.m.) were associated with a lower FEV1% predicted at evaluation, suggesting that age-specific thresholds might be warranted. Every increase in 1% time > 7.8 mmol/L at night was associated with a 0.76% lower predicted FEV1% [58]. Findings of key CGM outcome studies are presented in Table 2.

Although the evidence for the use of CGM as a complementary risk-assessment tool is increasing, large, long-term prospective studies assessing whether CGM measures can predict important CF outcomes are currently lacking. The ProspeC-F multicenter observational study is currently ongoing to evaluate whether CGM measures can predict clinical progression in patients with CF [44]. The primary aim is to identify which CGM measures at study inclusion are the most strongly associated with pulmonary function decline. The study will also investigate which CGM measures are the most strongly associated with other clinical markers, including nutritional status, CFRD diagnosis, and pulmonary exacerbations. The results of this study are eagerly awaited and will likely impact current CGM recommendations in this unique diabetes population.

## 5. Early Treatment of CGM-Detected Dysglycemia

Given the association of CFRD with increased morbidity and mortality, it is crucial to understand whether earlier diagnosis and treatment of CFRD, and potentially prediabetes, may mitigate pulmonary function decline and improve CF outcomes. In 2010, Hameed et al. reported that 4.5% of time > 7.8 mmol/L was associated with poor weight gain in children with CF and later found improved weight and lung function in those treated with insulin [57,59]. Frost et al. later reported improved pulmonary function using insulin in patients exceeding this threshold [56]. However, the threshold proposed by Hameed et al. was intended to predict insulin-remediable weight loss before the availability of highly effective modulator therapy. Indeed, many studies since then have found that many patients exceed this threshold, are not underweight, or do not meet CFRD criteria [46].

Gojsina et al. evaluated the impact of CFRD on pulmonary function and nutrition status using either an OGTT-defined CFRD threshold or a proposed CGM threshold (at least two peaks > 11.1 mmol/L and >10% time > 7.8 mmol/L). When insulin was initiated in patients with either OGTT-defined CFRD or CGM-defined CFRD, improved lung function and BMI Z-scores were observed, regardless of patient age [8]. Although these findings are encouraging, all the aforementioned studies were small and were not randomized trials.

The CF-IDEA (Cystic Fibrosis—Insulin Deficiency, Early Action) randomized controlled trial (ClinicalTrials.gov NCT01100892) was recently completed, with results pending, to determine whether starting insulin earlier than current practice (before CDRD) improves body weight and lung function in patients with CF. In this trial, patients with early glucose abnormalities based on frequently sampled OGTTs were randomized to once-daily insulin detemir for 12 months or to observation only [60]. If early treatment of dysglycemia is found to improve body weight and lung function, the importance of identifying and treating early dysglycemia, more often detected using CGM, will become even more apparent. Before a similar CGM-based interventional trial is conducted, large prospective studies are needed to establish which CGM measures, out of the many commonly reported, are most associated with clinical decline. This would then set the stage for CGM-based glycemic interventional trials aimed at slowing disease progression in patients with CF [51].

## 6. Future Directions

Large multicenter prospective studies are needed to determine the optimal thresholds of CGM measures for predicting clinically objective CF outcomes, including FEV1, BMI, and progression to CFRD, while also considering various clinical, epidemiological, genetic, and therapeutic factors. Large multicenter studies should evaluate CGM measures of hyperglycemia between 7.8 and 11.1 mmol/L, since studies have found associations between these values and clinical outcomes [22,29,42], with an emphasis on reporting cut-offs for %time metrics. It is still unclear whether single CGM measures or a combination of measures can predict clinically significant CF outcomes. Before employing CGM cut-off points for diabetes based on CF-specific outcomes, comparisons with reference standards, such as the OGTT, should be made to ensure alignment [47]. With the arrival of CFTR modulator therapies, the landscape of glucose abnormalities in CF is changing, challenging our understanding of CFRD. The effect of CFTR modulator therapy on CFRD evolution and glucose control is still unclear and needs further investigation. Prospective data collected under modulator therapy may provide insights into whether β-cell function improves with use. The rising prevalence of CFRD and chronic hyperglycemia will likely increase microvascular and conventional cardiovascular complications in patients with CF, and future studies may have to consider these outcomes as well [6].

## 7. Conclusions

In summary, while continuous glucose monitoring can offer valuable information about glucose dynamics in individuals with CF, at present, it still cannot replace the gold-standard OGTT for screening. Large long-term prospective studies are needed to determine which CGM measures and cut-offs best predict the clinical decline in CF and future CFRD risk. Furthermore, large-scale screening studies across multiple clinical phenotypes are needed to determine which CGM measures and cut-off points can reliably distinguish patients with and without CFRD. Although CGM cut-offs are not currently approved for CFRD diagnosis, the evidence supporting the use of CGM in diabetes screening and risk assessment is growing. For now, emphasis should be placed on increasing yearly OGTT screening, and intermittent CGM could be used alongside the OGTT, starting at 10 years of age. Future research should provide comprehensive CGM reporting, with an emphasis on %time metrics, alongside the gold-standard OGTT.

## Figures and Tables

**Table 1:** Findings of studies evaluating whether CMG can distinguish patients with and without cystic fibrosis-related diabetes [22,39].

First Author Publ. Year	Age Range	Patient Group Size	CGM Duration	CGM Used	CGM Blinded	Glycemic Categories at Baseline	CGM Measures Examined	Cut-Off Value/Findings
Chan et al., 2022 [39]	6–25	85	3–7 days	Medtronic, Minimed	Yes	NGT *n* = 27 AGT *n* = 45 CFRD *n* = 13	Mean sensor glucose, peak glucose %time > 7.8 mmol/L % time > 10.0 mmol/L %time > 11.1 mmol/L CV, MAGE	ROC AUC range of 0.43 to 0.57 for prediabetes ROC AUC range of 0.47 to 0.6 for CFRD %time > 7.8 mmol/L ROC AUC 0.60 (cut-off 9.7%, sensitivity 75%, specificity 55%)
Scully et al., 2022 [22]	8–70	77	25.5 ± 9 days	Free Style Libre	Yes	NGT *n* = 24 AGT *n* = 22 CFRD *n* = 31	AG % time 3.9–10.0 mmol/L % time 3.9–7.8 mmol/L % time < 3.9 mmol/L % of time < 3.0 mmol/L % time > 7.8 mmol/L % time > 10.0 mmol/L % of time > 13.9 mmol/L SD, CV, CONGA, MAGE	AG ROC AUC 0.92 (cut-off 6.2 mmol/L, sensitivity 87%, specificity 91%) SD ROC AUC 0.94 (cut-off 30.8, sensitivity 90%, specificity 91%) CV ROC AUC 0.88 (cut-off 30,6%, sensitivity 73%, specificity 86%) %time > 7.8 mmol/L ROC AUC 0.94 (cut-off 17.5%, sensitivity 87%, specificity 95%) %time > 10.0 mmol/L ROC AUC 0.94 (cut-off 3.4%, sensitivity 90%, specificity 95%) %time > 13.9 mmol/L ROC AUC 0.91 (cut-off 0.2%, sensitivity 83%, specificity 93%)

Abbreviations: CGM—continuous glucose monitoring, AG—average glucose, NGT—normal glucose tolerance, AGT—abnormal glucose tolerance, CFRD—cystic fibrosis-related diabetes, ROC—receiver operating characteristic, AUC—area under the curve, SD—standard deviation, CV—coefficient of variation, MAGE—mean amplitude of glycemic excursions, CONGA—continuous overall net glycemic action.

**Table 2:** Findings of studies exploring associations between CGM measures and CF outcomes [25,35,42,55,57,58].

First Author, Publ. Year, and Ref. No.	Age Range	Patient Group Size	CGM Duration	CGM Used	CGM Blinded	Cut-Off Value/Findings
Hameed et al., 2010 [57]	10–18	25	5 days	Medronic	No	≥4.5 %time > 7.8 mmol/L predicted decline in weight SD with 89% sensitivity and 86% specificity, ROC AUC 0.89, *p* = 0.003 ≥4.5 %time > 7.8 mmol/L detected a fall in %FVC of ≥3% over the preceding 12 months
Zorron et al., 2022 [35]	10–19	39	3 days	Medtronic MiniMed	Yes	No. of peaks/valleys (≥7.8, ≥11.1, <3 mmol/L) and %time (≥7.8, ≥11.1, <3 mmol/L) at baseline showed no associations with OGTT classification at follow-up (3.1 years) or FEV1 Peaks ≥ 7.8 mmol/L were associated with lower BMI
Elidottir et al., 2021 [55]	7–16	32	14 days	Freestyle Libre	No	Significant difference in FEV1% predicted in pts with ≥0.5 peaks > 11 mmol/L per day and those with <0.5 peaks per day (*p* = 0.018)
Leclercq et al., 2013 [25]	12–57	38	NR	Medronic	No	Decreased pulmonary function and an increased rate of Pseudomonas aeruginosa infections in pts with peaks ≥ 11.1 mmol/L
Taylor-Cousar 2016 [42]	20–65	18	3 days	Medronic		Glucose > 11.1 mmol/L on two dates predicted CFRD
Declercq et al., 2023 [58]	9–43	47	7 days	DexcomG4	Yes	AUC > 7.8 mmol/L and %time > 7.8 mmol/L during the night (10 pm-6 am) were associated with a lower predicted FEV1%

Abbreviations: CGM—continuous glucose monitoring, ROC—receiver operating characteristic, AUC—area under the curve, BMI—body mass index, FE1—forced expiratory volume in 1 s, NR—not reported.
